# Short Term Evaluation of an Anatomically Shaped Polycarbonate Urethane Total Meniscus Replacement in a Goat Model

**DOI:** 10.1371/journal.pone.0133138

**Published:** 2015-07-20

**Authors:** A. C. T. Vrancken, W. Madej, G. Hannink, N. Verdonschot, T. G. van Tienen, P. Buma

**Affiliations:** 1 Radboud University Medical Center, Radboud Institute for Health Sciences, Orthopaedic Research Lab, Nijmegen, The Netherlands; 2 Radboud University Medical Center, Radboud Institute for Molecular Life Sciences, Orthopaedic Research Lab, Nijmegen, The Netherlands; 3 Laboratory for Biomechanical Engineering, University of Twente, Enschede, The Netherlands; 4 Department of Orthopaedic Surgery, Via Sana Clinic, Mill, The Netherlands; Van Andel Institute, UNITED STATES

## Abstract

**Purpose:**

Since the treatment options for symptomatic total meniscectomy patients are still limited, an anatomically shaped, polycarbonate urethane (PCU), total meniscus replacement was developed. This study evaluates the *in vivo* performance of the implant in a goat model, with a specific focus on the implant location in the joint, geometrical integrity of the implant and the effect of the implant on synovial membrane and articular cartilage histopathological condition.

**Methods:**

The right medial meniscus of seven Saanen goats was replaced by the implant. Sham surgery (transection of the MCL, arthrotomy and MCL suturing) was performed in six animals. The contralateral knee joints of both groups served as control groups. After three months follow-up the following aspects of implant performance were evaluated: implant position, implant deformation and the histopathological condition of the synovium and cartilage.

**Results:**

Implant geometry was well maintained during the three month implantation period. No signs of PCU wear were found and the implant did not induce an inflammatory response in the knee joint. In all animals, implant fixation was compromised due to suture breakage, wear or elongation, likely causing the increase in extrusion observed in the implant group. Both the femoral cartilage and tibial cartilage in direct contact with the implant showed increased damage compared to the sham and sham-control groups.

**Conclusion:**

This study demonstrates that the novel, anatomically shaped PCU total meniscal replacement is biocompatible and resistant to three months of physiological loading. Failure of the fixation sutures may have increased implant mobility, which probably induced implant extrusion and potentially stimulated cartilage degeneration. Evidently, redesigning the fixation method is necessary. Future animal studies should evaluate the improved fixation method and compare implant performance to current treatment standards, such as allografts.

## Introduction

After (partial or total) meniscectomy the loads on the tibiofemoral cartilage surfaces substantially increase [1, 2]. Therefore, preservation of the meniscal tissue is prioritized during surgical treatment of meniscal lesions. Nevertheless, 94% of the meniscal injuries still requires (partial) meniscectomy [3]. Approximately half of the meniscectomized patients develop symptomatic osteoarthritis [4]. Meniscal allograft transplantation has been shown to be successful in reducing pain and functional limitations in symptomatic total meniscectomy patients [5, 6]. However, the supply of allograft menisci is limited and further challenged by the size-matching criteria that should be met to obtain a proper load distribution [7]. In addition, the integrity of the allograft tissue may be compromised by post-implantation shrinkage and retearing [8–10]. These limitations illustrate the need for an alternative treatment for total meniscectomy patients.

Efforts to develop a synthetic alternative to allograft menisci started several decades ago. In vivo tests with Teflon and Dacron meniscal substitutes resulted in wear particle induced synovitis and degenerative changes to the cartilage [11, 12]. More recently, a polyvinyl alcohol (PVA) hydrogel total meniscal replacement tested in a rabbit model for two years, showed good integrity and cartilage protection with respect to a meniscectomized joint [13]. However, after four months implantation in an ovine model more cartilage degeneration was observed in the PVA implant group than in the allograft group. Additionally, implant integrity was insufficient as complete radial tears were present in the posterior horns of all implants at the one year time point [14].

Its reported properties on biocompatibility, biostability, flexibility and wear resistance suggest that polycarbonate urethane (PCU) would be a suitable biomaterial for orthopaedic applications [15–17]. Furthermore, PCU is hydrophilic and should therefore be able to induce a fluid film between the bearing surfaces, mimicking the native lubrication mechanism in synovial joints. Together, these characteristics suggest that a PCU implant could act as a potential substitute to the native meniscus, which was confirmed in a six month ovine animal experiment showing cartilage histopathological scores to be similar for an anatomically shaped PCU total medial meniscus replacement and the non-operated control group [18]. However, drastic changes to the geometry and fixation of the implant were implemented, resulting in a non-anatomical, disc-shaped, free-floating implant for use in humans [19]. In addition, this implant requires a functional peripheral rim of the meniscus, which excludes its use in total meniscectomy patients [20].

Stimulated by the promising results previously obtained with PCU implants in articular joints, we have designed an anatomically shaped, PCU, total meniscus replacement. To obtain initial insights into the in vivo performance of this novel meniscus replacement, the current study describes a three month goat trial. Specifically, we assessed the implant location in the joint, its integrity in response to physiological loading and the effect of the implant on the histopathological condition of the synovial membrane and the articular cartilage.

## Materials and Methods

### Ethics statement

This study was carried out according to the demands of the Dutch Experiments on Animals Act and the guidelines of the Federation of European Laboratory Animal Science Associations. The study protocol was approved by the institutional Animal Ethics Committee of the Radboud University Nijmegen, The Netherlands (RU-DEC 2012–155).

### Implant

**Fig 1:**
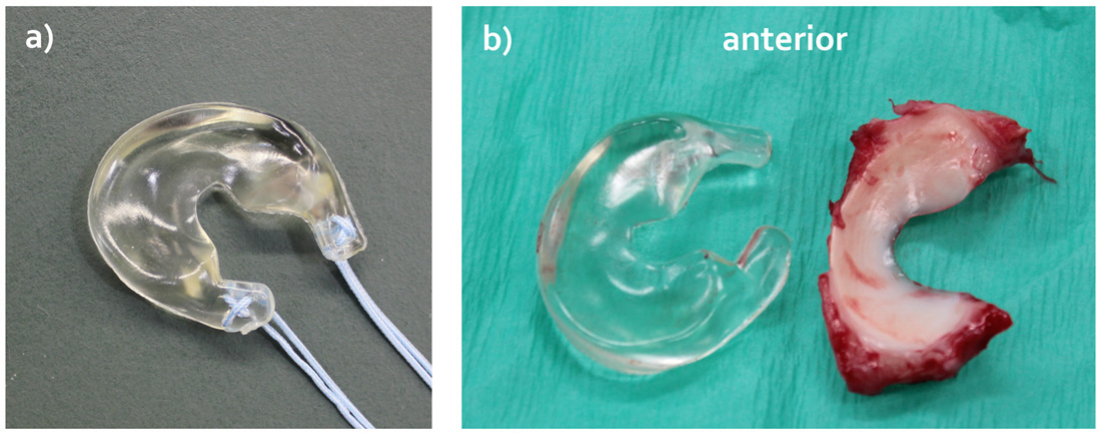
Goat version of the anatomically shaped, polycarbonate urethane (PCU) total medial meniscus replacement. a) Implant including the sutures for fixation to the tibia plateau. b) Direct comparison between the goat PCU implant and a native medial meniscus that was replaced by the implant.

Implant geometry was derived from an excised medial meniscus of a female Dutch milk goat (Capra Hircus Sana) that was soaked in Omnipaque Iohexol (300 mg I/mL, GE Healthcare, Inc., Princeton, NJ, USA) for 2 hours and subsequently microCT scanned in air (μCT 40, Scanco Medical AG, Brüttisellen, Switzerland). The scans were taken using the following settings: 70 kVp, 114 μA, 300 ms exposure time, 264 slices and 36x36x36 μm resolution. A 3D model of this meniscus was created and the meniscal horns were extended five millimeters to allow some flexibility in adjusting the width of the implant to that of the native meniscus and for suture fixation of the implant to the tibia plateau. Polycarbonate urethane (PCU, Bionate II 80A, DSM Biomedical, Berkeley, CA) implants were subsequently produced by injection molding (Fig 1a). All implants were sterilized by ethylene oxide gas (Synergy Health, Venlo, the Netherlands).

### Study design

Thirteen female Dutch milk goats (Capra Hircus Sana) were included in the experiment. Since the implant was available in only one size, the animals were assigned to the experimental groups based on their weight. The weight of the implant group animals was matched to that of the animal whose meniscus served as a model for implant geometry. In seven animals (age 29.6±5.4 months, weight 65.9±5.0 kg) the medial meniscus of the right stifle joint was replaced by the PCU implant (implant group (I)). Six goats (age 38.3±13.0 months, weight 66.8±9.6 kg) were subjected to a sham surgery (sham group (S)). The non-operated left stifle joints served as control joints, for which a distinction was made between the implant-control group (I-c) and the sham-control (S-c) group. Three months post-surgery, the animals were sacrificed and the knee joints were collected for radiological, macroscopic and histological evaluation.

The sample size for our study was based on the results of a previous study on meniscal transplantation in an ovine model [21]. A sample size calculation showed that six animals per group would be required to detect a difference in Mankin score of four points with a standard deviation of two points with a power of 80% and a significance level of α = 0.05. To be able to cope with the potential loss of an animal due to complications of the meniscal replacement surgery, we decided to include one extra animal in the implant group.

### Surgical procedure

The goats were anaesthetized by intravenous administration of propofol (4 mg/kg) and subsequently intubated. Anesthesia was maintained using a mixture of nitrous oxide, oxygen and 1.5% isoflurane. The animals were placed in supine position, and the medial compartment of the stifle was accessed through an arthrotomy and subsequently the medial collateral ligament (MCL) and synovial membrane were transected. The medial meniscus was circumferentially separated from the synovial membrane and a medial meniscectomy was performed by sharp dissection of the anterior and posterior menisco-tibial ligaments. Because of the variation in joint and native meniscus size, the implant fit was evaluated and if necessary the horn extensions were trimmed to match the width of the native meniscus as closely as possible. Using an Acufex Protrac drill-guide (Smith&Nephew, Andorver, MA, USA), two transosseous tunnels (2.5 mm diameter) were drilled from the anterior part of the proximal tibia to the anterior and posterior attachment sites of the meniscal horns on the tibial plateau. Both implant horns were supplemented with FiberWire No. 2 sutures (Arthrex Inc, Naples, FL, USA) (Fig 1a), which were guided through the bone tunnels and knotted together on the anteromedial side of the tibia to fix the implant. The MCL was repaired using FiberWire No. 2 sutures. The capsule, fascia and skin were closed in layers using resorbable Vicryl 2–0 sutures (Ethicon, Sommerville, NJ, USA). The animals in the sham group underwent the same procedure, except for the steps related to replacement of the meniscus (meniscectomy, bone tunnel drilling and implantation of the prosthesis). Post-operatively, analgesics were administered by intramuscular injection (buprenorphine 0.3 mg/ml, two times 1 ml/12 hours and flunixine 50 mg/ml, three times 1.2 ml/24 hours). Antibiotics (ampicilline, two times 15 mg/kg/48 hours) were injected subcutaneously. To promote the wound healing process, the animals were placed in a custom-designed hammock for six to ten days following the surgery. The hammock allowed voluntary load bearing of the operated leg, but restricted mobility. Hereafter, the goats were housed in a large stable and allowed to freely load the operated leg. After three months follow-up, the goats were killed by intravenous injection of an overdose of sodium pentobarbital. Thereafter, the femur was transected close to the femoral head, leaving the skin and soft tissues around the stifle joint intact.

### MR imaging and extrusion measurements

All limbs of the implant and sham groups were imaged on a 7 Tesla small animal MRI scanner (ClinScan, Bruker, Billerica, MA, USA), using a 3D DESS sequence. The scanning parameters were: repetition time 10 ms, echo time 2.91 ms, flip angle 20°, slice thickness 0.29 mm, no interslice gap, a voxel size of 0.29x0.29 mm and a field of view 129x129 mm. Tibial and femoral extrusion of the implant and the native medial meniscus in the sham group were evaluated from the MR images following the definitions by Verdonk et al. [22]. Measurements were taken in the mid-coronal plane, displaying both tibial intercondyllar eminences.

### Tissue dissection and macroscopic evaluation

**Fig 2:**
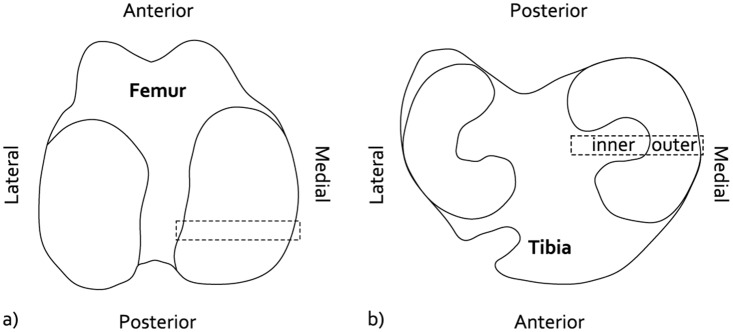
Osteochondral specimens to evaluate cartilage histopathological condition. a) Location of the femur specimens. b) Location of the tibia specimens, displaying the in the inner and outer regions that were separately scored.

Following MR imaging, the stifle joints were carefully dissected. First, the MCL repair was macroscopically inspected. Then, the synovium was opened and macroscopically imaged under standardized lighting conditions. Samples from the synovial membrane covering the infrapatellar fat pad were harvested for histological analysis. The femoral and tibial articular cartilage surfaces were macroscopically imaged under standardized lighting conditions before and after India ink staining. Undiluted India ink (Rotring International GmbH & Co, Hamburg, Germany) was applied for 30 seconds and rinsed with tap water for 30 seconds. Approximately 5 mm wide, radially oriented osteochondral specimen from the central regions of the medial tibia plateau and medial femur condyle were harvested for histological analysis (Fig 2).

Gross appearance of the India ink stained articular cartilage surfaces was blindly assessed from the macroscopic images by two independent observers (ACTV & PB) applying the scoring scheme recommended by the OARSI histopathology initiative [23]. This score assesses changes related to tissue morphology (presence of roughening, fibrillation, fissures or complete erosion).

### Histology

The synovial specimen were fixed in 4% neutral phosphate buffered formaldehyde for 48 hours and subsequently embedded in paraffin and 5 μm thick sections were cut. The sections were stained with haematoxylin and eosin (HE) and scored [23]. Since some differences were observed between the synovial of the implant and sham groups, additional histochemical staining of these sections was performed to identify whether these changes could be explained from the natural wound healing process. To indicate activated macrophages, the sections were stained with acid phosphatase [24]. To detect whether observed debris contained iron from blood degradation products, a Perl’s Prusian blue staining was performed [24]. All synovium sections were assessed by conventional and polarized light microscopy.

The osteochondral specimens were fixed in 4% neutral phosphate buffered formaldehyde for 24 hours and subsequently decalcified in 10% PVP-EDTA. The tissue blocks were embedded in paraffin and 5 μm thick sections were cut. The sections were stained with Safranin-O / Fast Green. Cartilage histopathological condition was blindly scored by two independent observers (ACTV & PB) according to a modified Mankin scoring scheme [23]. The surface area affected by structural degenerative changes below 10% of the cartilage thickness was separately scored (Affected Area Score), ranging from 0 (no structural damage) to 5 (>75% of the surface area affected by structural changes). Because of large differences in histopathological condition, separate scores were assigned to the inner and outer halves of the tibia sections (Fig 2b).

### Implant deformation

**Fig 3:**
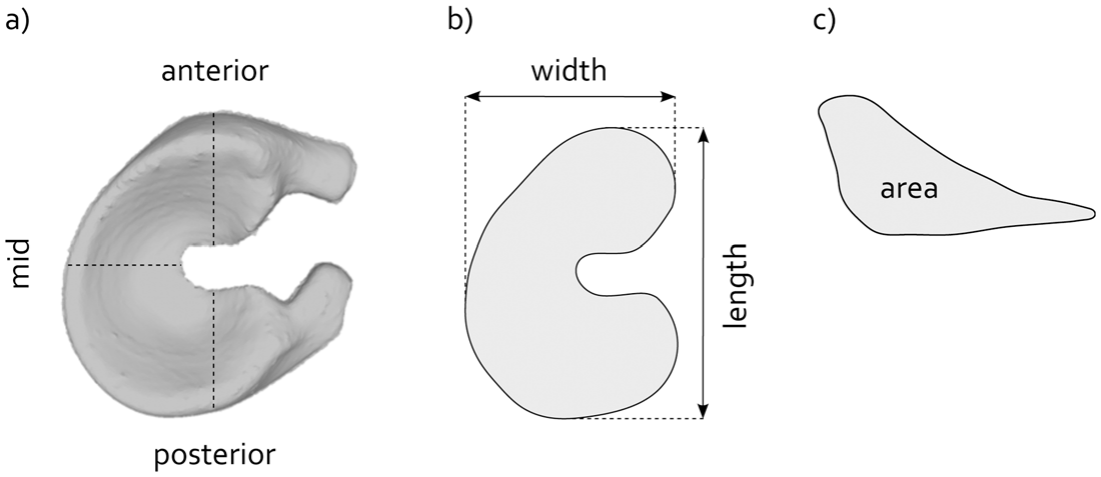
Evaluation of implant deformation. a) A 3D model of the implant, illustrating the locations of the cross-sections through the anterior and posterior horn and mid regions. b) Projection of the implant onto the transverse plane, including the definitions of implant length and width. c) Example of a cross-section, including the definition of the cross-sectional area.

All explanted prostheses were stored at room temperature in 0.15 M phosphate buffered saline for a minimum of four weeks. A non-implanted reference prosthesis was kept under similar conditions for four weeks. The implants were microCT scanned in air using the same settings as described in the ‘implant’ section of this paper. The images were segmented using Mimics software (v14.0, Materialise, Leuven, Belgium) and 3D models of the implant geometries were created. To be able to compare corresponding regions for each implant, the 3D models were aligned by a Hausdorff distance minimization algorithm [25]. Length and width of each implant were determined from the projection on to the transverse plane. As the horn geometry was potentially different for each implant, the horns were excluded from the analysis (Fig 3a and 3b). In addition, the height, width and area of cross-sections through the anterior and posterior horn and mid region of the implant were quantified (Fig 3b and 3c).

### Statistical analysis

The scores of the two independent observers were averaged preceding statistical analysis. Since the synovium and cartilage scores were not normally distributed, non-parametric tests were performed. Paired data (sham versus sham-control and implant versus implant-control) were analyzed using Wilcoxon signed rank tests. Independent data sets (implant versus sham, implant versus sham-control and implant-control versus sham-control) were evaluated using Mann-Whitney U tests. The extrusion data were analyzed using an independent-samples t-test. Differences between groups were considered significant for p-values smaller than 0.05. All statistical analyses were performed using SPSS Statistics (v20, IBM SPSS Statistics, Armonk, NY, USA).

## Results

### General findings

All goats tolerated the surgery well. Initial malpositioning of the bone tunnel occurred in three animals (two times anterior, one time posterior), resulting in full-depth cartilage defects. The length of the implant corresponded well to that of all native menisci that were replaced (Fig 1b). Six implants were trimmed to match the width of the native meniscus. The wound healing process was without complications and all animals could be released from the hammock within ten days post-surgery. The goats returned to normal activity levels within two weeks. Any initial adaptations of the animal’s gait had normalized six weeks post-operatively. No signs of distress were observed during the three month follow-up period.

### Implant position in the knee joint

**Fig 4:**
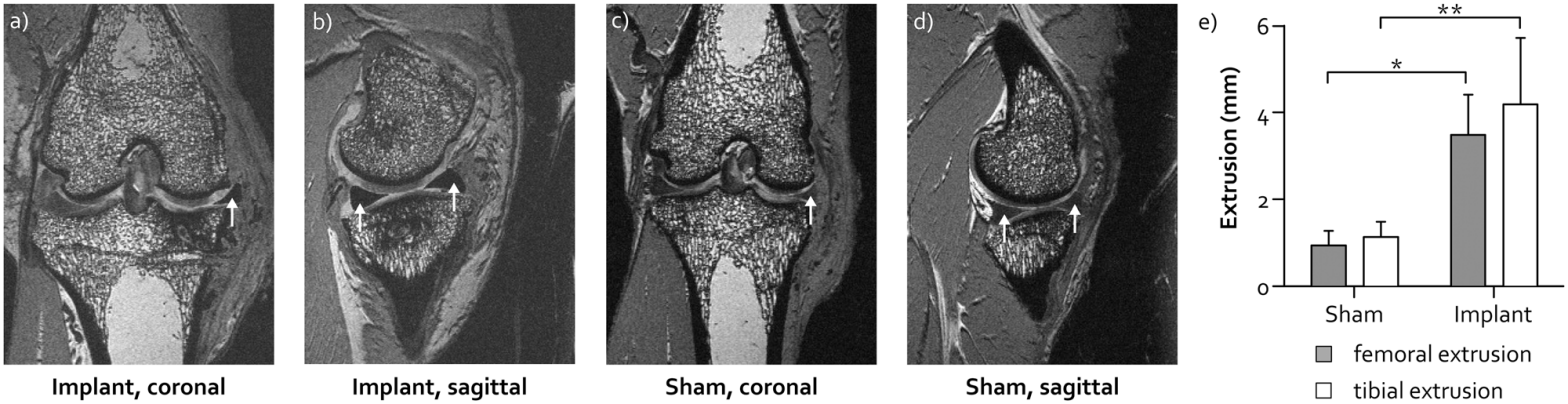
Position of the implant in the joint. Representative MR images of a knee joint with the PCU implant (a and b) and the native medial meniscus in the sham group (c and d). The white arrows indicate the location of the implant (in a and b) or the native meniscus (in c and d). e) Extrusion (mean ± SD) of the native medial meniscus in the sham group and the implant, with respect to the femur (white bars) and tibia (grey bars). * refers to p<0.001, ** to p = 0.002.

The sagittal plane MR images showed good conformity between the implant and the femoral and tibial cartilage surfaces (Fig 4a). The position of the implant horns on the tibia plateau corresponded well to that of the native meniscus horns (Fig 4a and 4c). In the mid-coronal plane, the mid body of the implant was pushed more outside the joint space than the native meniscus (Fig 4b and 4d). Quantitative assessment showed a difference of 2.5 mm in tibial extrusion (p<0.001) and 3.0 mm in femoral extrusion between the implant and sham groups (p<0.001 and p = 0.002 respectively, Fig 4e). The degree of extrusion could not be related to rupture of the posterior fixation suture which was observed for several implants.

### Macroscopic observations

The medial aspect of all operated knee joints showed thickening due to fibrosis of the soft tissues surrounding the repaired MCL. Regional extracapsular ossification of the scar tissue was observed in one animal from the implant group. The synovial fluid was of normal consistency and color in all animals. In one joint of the implant group severe discoloration of the synovial membrane was observed, however, generally the macroscopic changes to the synovial membrane were minimal.

Macroscopic inspection revealed that all implants were intact. However, integrity of the fixation sutures was always compromised by elongation or wear or breakage. In two animals both posterior fixation sutures were torn at the implant-bone interface. In three other goats, one out of two posterior fixation sutures was torn at the same location. The posterior sutures always showed signs of wear, compromising the surface layer and part of the deeper layers of the suture material.

**Fig 5:**
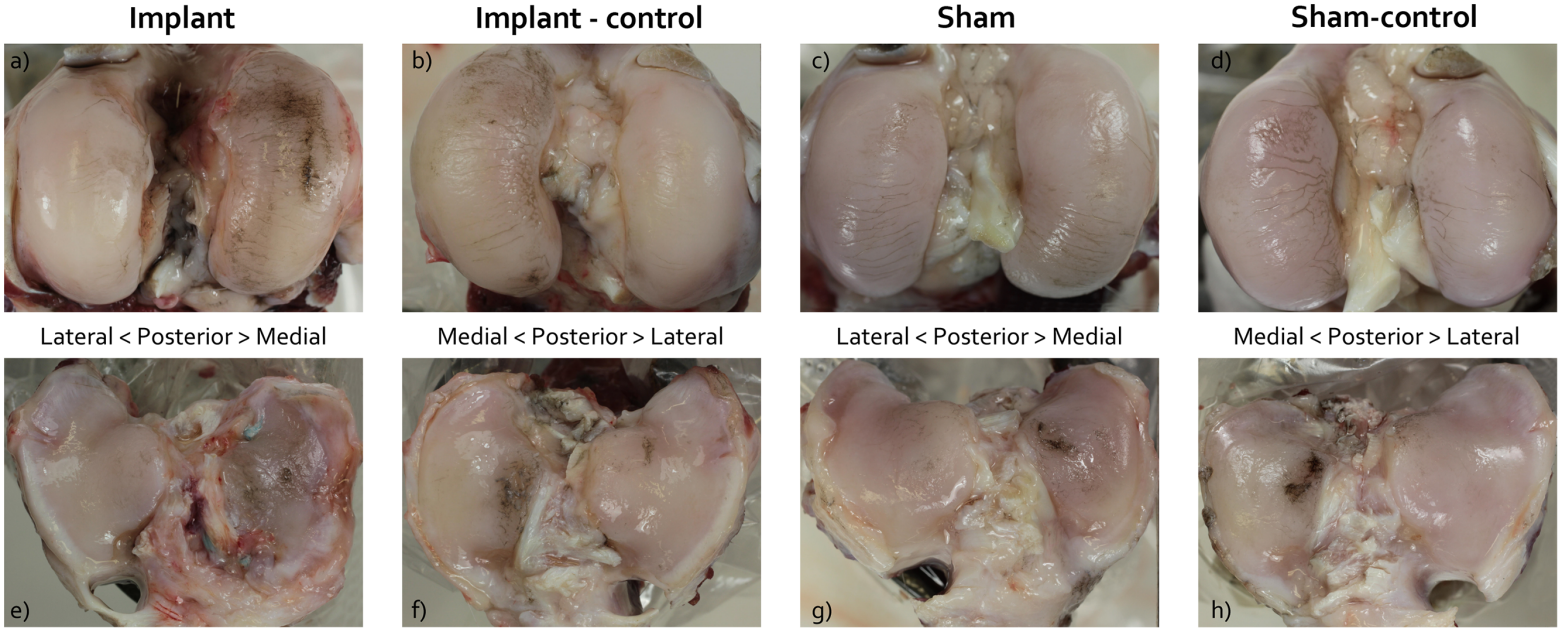
Macroscopic images of the india ink stained cartilage surfaces. (a-d) femur condyles and (e-h) tibia plateaus. Figs a-d show the presence of femoral condyle cartilage fibrillation in all experimental groups (white arrows), while Figs e-f show focal india ink staining of the central tibia plateau for all groups (black arrows). The femur and tibia of the implant group (a and e) showed more extensive ink staining than the other groups.

Signs of cartilage degeneration were present in all operated as well as in all non-operated joints. All femoral condyles showed marked fibrillation along the mediolateral axis, which was most pronounced on the inner aspect of the medial condyle (Fig 5a–5d). This resulted in highly similar macroscopic femoral cartilage scores for all groups (Median (IQR): Implant: 2.00 (0.00); Implant-control: 2.00 (0.00); Sham: 2.00 (0.75); Sham-control: 2.00 (0.00)). A majority of the joints of the implant group showed damage patterns along the anteroposterior axis on the medial femur condyles (Fig 5a). The cartilage covering the central region of the medial tibia plateau was also damaged in all groups (Fig 5e–5h). The medial tibia plateau of the implant group showed additional fibrillation. In two animals of the implant group cartilage erosions down to the subchondral bone were found. However, the location of these chondral defects corresponded to the location of the malpositioned bone tunnels. The median (IQR) macroscopic scores for the tibial cartilage were: Implant: 3.00 (0.75); Implant-control: 2.00 (1.00); Sham: 1.25 (0.88); Sham-control: 2.00 (0.00)). Significant differences were found between the scores of the implant and sham, and implant and sham-control groups (p = 0.004 and p = 0.012 respectively).

### Histological observations

**Fig 6:**
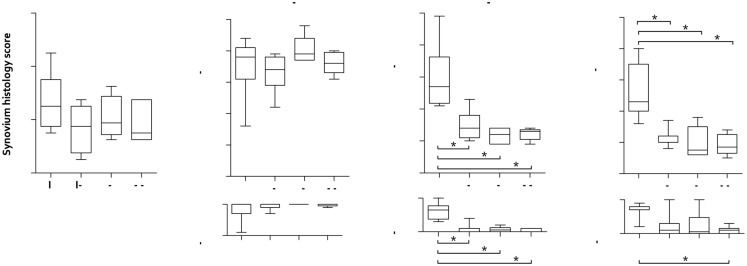
Synovium and cartilage histopathological scores represented as box plots. a) Synovium score. b) Inner tibia cartilage score. c) Outer tibia cartilage score. d) Femur cartilage score. The cartilage histopathological scores are split into the modified Mankin and Affected Area scores, representing respectively the structural/morphological condition of the cartilage and the extent of potential damage. I = Implant; I-c = Implant-control; S = Sham; S-c = Sham- control. The box extends from the 25^th^ and 75^th^ percentile and shows the median as a horizontal line crossing the box. The whiskers represent the minimum and maximum scores. * refers to a significant difference between two experimental groups.

**Fig 7:**
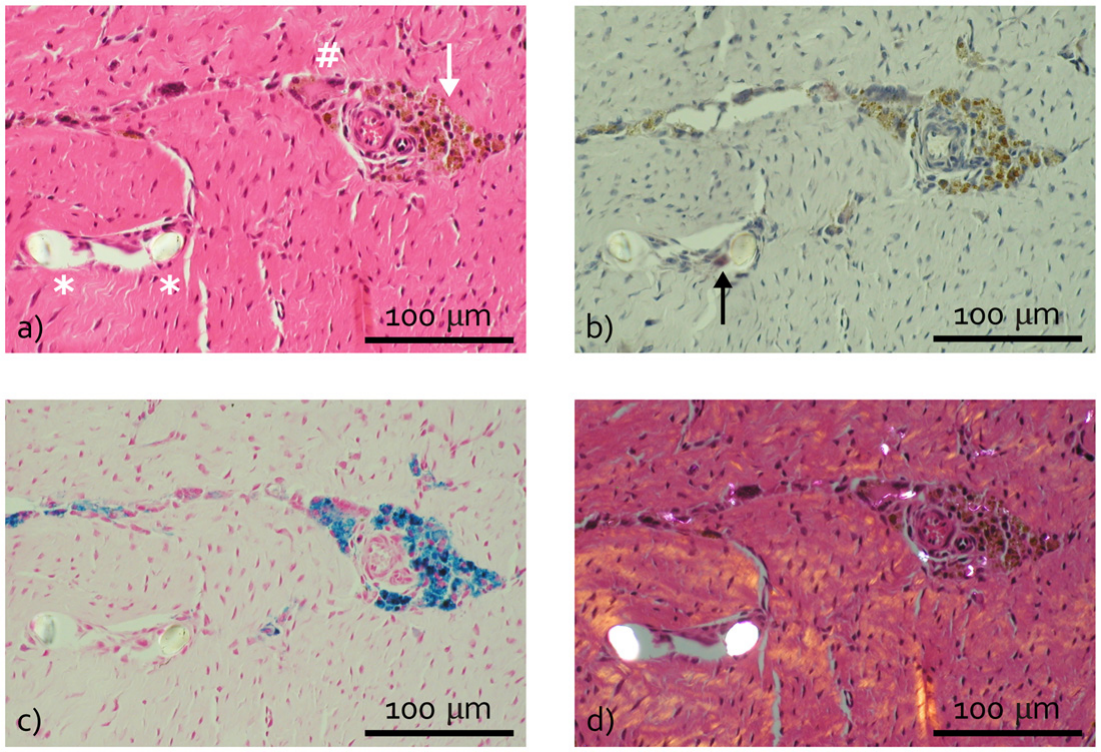
Typical examples of synovium histology of the implant group. a) Haematoxylin eosin (HE) stained section showing macrophages (white arrow) and a giant cell (#) containing brown debris and two fibrous fragment cross-sections with a diameter of approximately 20 μm (*) surrounded by a giant cell. b) Acid phosphatase section showing weak positive staining of the fiber-surrounding giant cell (black arrow). c) Perl’s Prussian blue section, which is positive at the location of the brown debris visible in a) and b). d) Polarized light HE section showing birefringent fragments in the occasional macrophages and giant cells.

Signs of intimal hyperplasia, subintimal fibrosis and an increased vascularity of the synovial membrane were observed in all experimental groups, resulting in similar synovium histology scores (Fig 6a). Local infiltration of lymphocytes was only observed for the implant group, but lymphoid aggregates were absent. In several synovium HE sections from the implant group the area of sub-intimal fibrosis contained a small numbers of macrophages and giant cells, which contained brown debris (Fig 7a). While the acid phosphatase staining was weak or negative for these cells (Fig 7b), the debris stained intensely in response to the Perl’s Prussian blue staining (Fig 7c). This positive staining for iron suggests that these cells were recruited to phagocytize blood containing debris. As this observation was restricted to the implant group it cannot be linked to the arthrotomy but is most likely caused by drilling the bone tunnels. Additionally, occasional giant cells were observed in the implant group, which contained fibrous material with a diameter of maximal 20 μm. Polarized light microscopy showed the birefringence of these fibrous fragments (Fig 7d). The birefringent nature and diameter of these fibers indicate that they originate from the suture material used for implant fixation.

**Fig 8:**
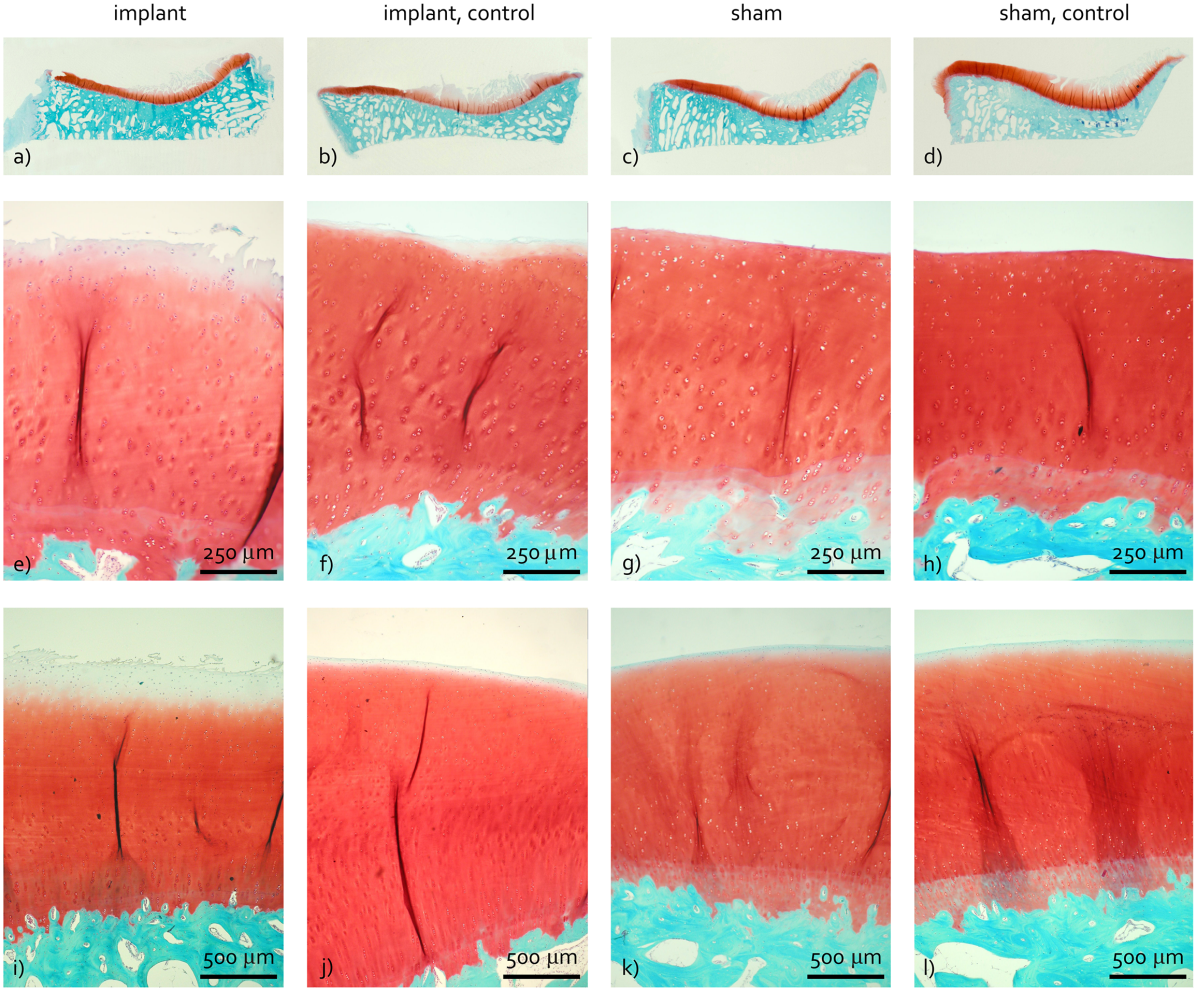
Representative examples of cartilage histology. a-d) Tibia sections. e-h) Magnifications of the outer tibia region. i-l) Femur sections. Images a-d show severe damage to the cartilage of the inner tibia plateau for all four experimental groups. The cartilage of the outer tibia and femur of the implant group (e and i) showed structural damage and loss of Safranin-O staining of the surface layer, while this was absent for the other experimental groups.

Due to difficulties during the preparation of the tibia cartilage histological samples, one specimen of the implant, sham and sham-control groups was missing for histological analysis. The cartilage of the inner aspect of the tibia plateau displayed severe degenerative changes in practically all operated and non-operated joints. These areas showed loss of structural integrity as a result of deep fibrillation of the cartilage. Also, the amount of Safranin-O staining was considerably reduced (Fig 8a–8d). Consequently, the histopathological scores for the inner tibia plateau were consistently high for all experimental groups (Fig 6b).

The tibial cartilage in direct contact with the implant showed signs of fissuring, cell cloning and loss of Safranin-O staining, which were limited for the other groups (Fig 8e–8h). This resulted in significant differences in modified Mankin scores between the implant and implant-control groups (p = 0.027), the implant and sham groups (p = 0.004) and implant and sham-control groups (p = 0.006, Fig 6c). Similar changes were observed for the cartilage of the femur condyle (Fig 8i–8l), resulting in significant differences in modified Mankin scores between the implant and implant-control groups (p = 0.018), the implant and sham groups (p = 0.004) and implant and sham-control groups (p = 0.004, Fig 6d). The Affected Area Score of the outer tibia cartilage was larger for the implant group compared to the implant-control, sham and sham-control groups (Fig 6c, p = 0.046, p = 0.004 and p = 0.005 respectively). Morphological damage to the femoral cartilage was more extended for the implant group with respect to the sham-control group (Fig 6d, p = 0.004).

### Implant deformation

**Fig 9:**
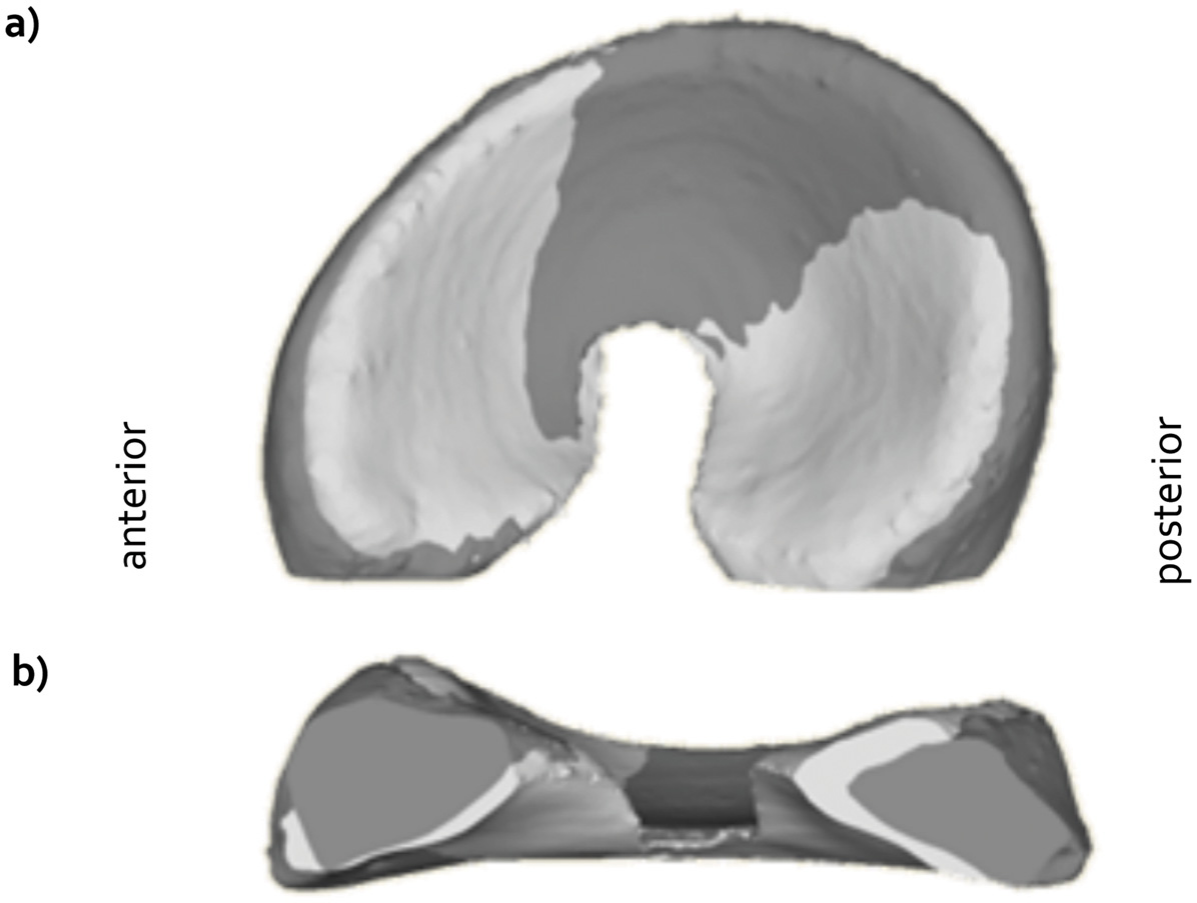
Representative example of implant deformation. a) Top view. b) Side view). The light grey color is the reference prosthesis, the dark color the explanted prosthesis.

Gross implant geometry was maintained during the three month follow-up period. Inspection of the microCT scans showed that the fixation sutures did not damage the implant bulk material. In the transverse plane, implant deformation was characterized by slight lengthening along the anteroposterior axis (Fig 9a and 9b, Table 1). Deformation of the anterior horn and mid body was minimal, while the posterior horn cross-sectional area decreased with 12.33% (Table 1).

**Table 1:** Change in implant dimensions over the three month implantation period with respect to a non-implanted reference implant.

	Difference with respect to the reference implant	
	Mean	SD
Length	2.57%	0.71%
Width	0.75%	0.71%
Anterior area	-1.82%	2.16%
Mid area	-1.12%	2.93%
Posterior area	-12.33%	2.27%

## Discussion

This three month study in goats was designed to provide basic insights into the in vivo performance of a novel PCU meniscal implant, with a specific focus on implant (fixation) integrity and potential changes to the synovium and cartilage histopathological condition. The results show that integrity of the implant bulk material was satisfactory as the shape of the implant was well maintained after three months of physiological loading. The implant fixation on the other hand, could not withstand physiological loading in the goat knee, resulting in extrusion of the implant. The meniscal replacement did not induce an inflammatory response in the goat knee joint. However, the onset of degenerative changes to the articular cartilage was observed in the implant group, while these were absent for experimental groups where the native meniscus was in place.

The MR images demonstrated that the implants were well positioned in the sagittal plane. However, in the coronal plane, the implants showed a substantially higher degree of extrusion than the native menisci. Because extrusion reduces the contact area between the meniscal replacement and the articulating cartilage, it may increase the loads on the cartilage. As mechanical overloading of the cartilage has been associated with the development of osteoarthritis, extrusion of any meniscal replacement should be minimized. Nevertheless, despite the frequent observation of extrusion after human meniscal allograft transplantation, no adverse effects on clinical outcomes were detected [26–28]. The origin of the extrusion can be threefold. Firstly, failure of the suture fixation allowed the implant to be pushed out of the joint. Secondly, the implant may have been elongated as a result of the hoop stresses acting on the material. However, as the width of the implant showed minimal change over the three month implantation period (Table 1), this explanation is unlikely. Thirdly, the implant body was slightly wider than most native menisci (Fig 1b), which could not always sufficiently be corrected by shortening of the horn extensions. Probably, the observed implant extrusion originated from a combination of cause one and three.

The implant bulk material showed to be compliant and gross implant geometry was maintained throughout the period of implantation. The MR images indicate that a good congruency was obtained between the implant and the femoral cartilage. Minimal lengthening of the implant along the anteroposterior axis was probably induced to adapt to the femoral geometry during flexion. A biomechanical study in an ovine model showed that the highest loads occur in the posterior aspect of the sheep knee [29]. Because of the close anatomical correspondence between the goat and sheep knee [30], it can be expected that similar trends can be observed in the goat knee. This explains the larger implant deformation in the posterior region compared to the mid and anterior regions. As implant deformation was monitored several weeks post-mortem, it can be classified as permanent deformation. It is unclear whether this deformation process was relatively abrupt or of continuous origin. Prolonged implantation periods are necessary to evaluate long-term implant integrity.

The present study confirms previous results on the good biocompatibility of PCU implants in synovial joints [15, 16, 18]. We did not observe evidence of an infection or immunological response caused by the implant material. The correspondence in synovium histopathology scores for all experimental groups indicates that the mild changes to the synovium were rather a characteristic of the goat model than of the intervention studied here. The occasional and localized presence of macrophages and giant cells observed in the implant group were associated with suture wear fragments and debris caused by drilling the bone tunnels.

Goats and sheep are considered suitable models for evaluating the effects of meniscal substitution on the articular cartilage because they rapidly develop osteoarthritic changes following (partial) meniscectomy or meniscal replacement [14, 21, 31–33]. However, all four groups in our study showed severe degenerative changes of the articular cartilage of the inner aspect of the medial tibia. This is a strong indication that the goat knee also tends to develop spontaneous osteoarthritis. Similar observations of spontaneous osteoarthritis of the unprotected area of the medial tibial cartilage in goats as young as two years old were also reported by Little et al. [23]. Also in other common models used within meniscal replacement research, the dog and sheep model, spontaneous degeneration of the knee articular cartilage was observed [18, 31, 34–36]. These findings complicated the assessment of the potential change in load induced by the meniscal substitute in the highly loaded central region of the tibia plateau, as the cartilage modified Mankin scores of the experimental and control groups were both equally high. By separately scoring the inner and outer regions of the tibial cartilage sections, however, we were still able to assess the direct influence of the implant on the articular cartilage condition.

Histological analysis of the articular cartilage revealed more degeneration in the implant group compared to the control groups, reflected by more severe histomorphological changes and a larger extent of structural damage of the femoral and outer tibia cartilage. The damage running along the anteroposterior axis of the medial femur condyle was only visible on the macroscopic images of the implant group, indicating specific involvement of the implant in the development of these patterns. Cartilage damage was most severe in the two goats in which the posterior fixation sutures ruptured. It has been shown that without fixation of the horns, the pressure distribution under a meniscal allograft closely resembles that after meniscectomy [37, 38]. Thus, the loss of fixation in our experiment has presumably resulted in a detrimental change of the contact pressures and thereby may have aggravated cartilage degeneration. Because the fixation integrity was affected for the remaining implants as well, these effects may have affected the cartilage histology scores in all animals of the implant group.

Previous animal studies, evaluating both permanent and tissue regenerating total meniscal replacements, also showed more severe cartilage damage in the implanted group compared to the sham or non-operated controls [14, 39, 40]. The damage was attributed to the surface characteristics of the biomaterial [40] and excessive motion due to inadequate circumferential fixation [14]. Contrary to our findings, Zur et al. reported only minor changes to the cartilage when evaluating a PCU meniscal replacement in sheep. However, care should be taken to extrapolate these results because of their small experimental group size. The difference in outcomes between their study and the present study is presumably caused by the complications regarding the implant fixation that were experienced in our study.

Failure of the double-thread suture fixation strategy within the three month implantation period was unforeseen. For meniscal allograft transplantation in human patients, the soft tissue fixation technique with non-resorbable sutures is common practice [6], and no differences in clinical outcomes have been observed between grafts fixed with bone plugs or with the suture-only technique [27]. In addition to horn fixation, allograft menisci are generally circumferentially fixed to the knee capsule [41]. However, biomechanical studies have shown that the circumferential fixation does not play a role in the functional performance of a meniscal graft [42, 43]. These observations, combined with the surgical complexity of the circumferential fixation step, have resulted in our decision to restrict the fixation to the horns only. As literature does not report on suture breakage at the bone-graft interface after long term follow-up of allograft patients, the occurrence of fixation failure in our experiments indicate that circumferential fixation may shield the horn fixation sutures from rubbing against the sharp edge of the bone tunnel. Evidently, it is essential to improve the fixation strategy of our implant for future applications.

This short-term study was performed to obtain initial experience with the in vivo performance of our novel meniscal implant. Consequently, only two surgical groups, an implant and a sham-surgery group, were included. Previous studies evaluating meniscal allograft transplantation in large animal models reported on considerable damage to the articular cartilage [14, 21, 44], and a chondro-protective effect has not been proven in humans either [5, 6]. Nevertheless, clinically allografts are considered effective in relieving pain and improving function for total meniscectomy patients [5, 6]. For future long-term experiments it is therefore necessary to included additional comparisons with both a total meniscectomy group and an allograft transplantation group, in order to predict the clinical value of the novel implant.

## Conclusion

This study showed that the novel, anatomically-shaped PCU implant for total meniscal replacement is biocompatible and that the implant bulk material can withstand repetitive physiological loading in a large animal model. Failure of the implant fixation likely increased implant extrusion from the knee joint, thereby potentially increasing implant mobility. This may have contributed to the development of cartilage damage as observed in this study. Apart from improvements to the fixation strategy, future tests require a direct comparison of the implant with an allograft and a total meniscectomy group, to be able to extrapolate performance of the implant to the current clinical practice.

## Supporting Information

S1 Checklist Completed ARRIVE Guidelines Checklist. (PDF)

S1 Table Individual scores and outcomes per knee joint. (I) Cartilage macroscopic score, (II) Implant extrusion, (III) Implant deformation, (IV) Synovium histology score, (V) Cartilage Modified Mankin Score and (VI) Cartilage Affected Area Score.
(PDF)
