# Correction: Short Term Evaluation of an Anatomically Shaped Polycarbonate Urethane Total Meniscus Replacement in a Goat Model

**DOI:** 10.1371/journal.pone.0137936

**Published:** 2015-09-03

**Authors:** The PLOS ONE Staff

Fig 6, “Synovium and cartilage histopathological scores represented as box plots”, appears incorrectly due to an error introduced during the typesetting process. The publisher apologizes for this error. Please see the corrected Fig 6 here.

**Fig 6:**
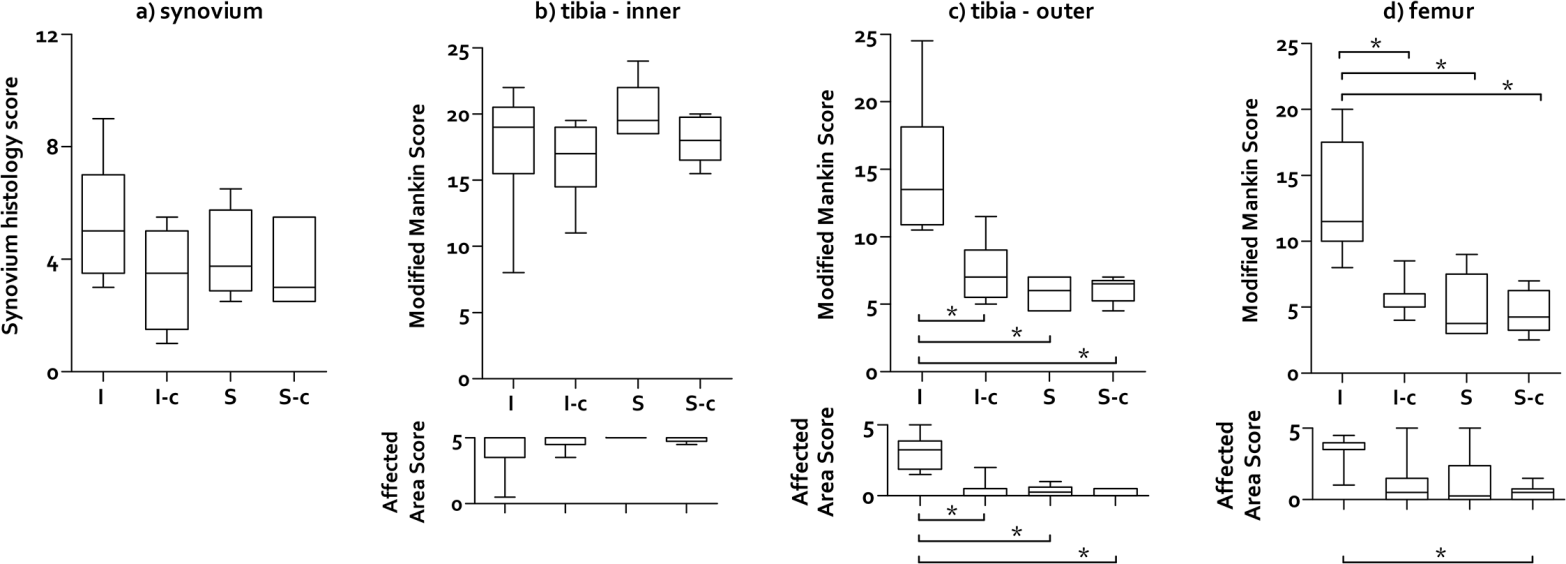
Synovium and cartilage histopathological scores represented as box plots. a) Synovium score. b) Inner tibia cartilage score. c) Outer tibia cartilage score. d) Femur cartilage score. The cartilage histopathological scores are split into the modified Mankin and Affected Area scores, representing respectively the structural/morphological condition of the cartilage and the extent of potential damage. I = Implant; I-c = Implant-control; S = Sham; S-c = Sham- control. The box extends from the 25^th^ and 75^th^ percentile and shows the median as a horizontal line crossing the box. The whiskers represent the minimum and maximum scores. * refers to a significant difference between two experimental groups.
