# Supplementary material for: Short Term Evaluation of an Anatomically Shaped Polycarbonate Urethane Total Meniscus Replacement in a Goat Model
Source: PLoS One. 2015 Jul 20;10(7):e0133138. doi: 10.1371/journal.pone.0133138 (PMC4507942; doi:10.1371/journal.pone.0133138)
Supplement: S1 Table — (I) Cartilage macroscopic score, (II) Implant extrusion, (III) Implant deformation, (IV) Synovium histology score, (V) Cartilage Modified Mankin Score and (VI) Cartilage Affected Area Score. (PDF) [file pone.0133138.s002.pdf]

	Cartilage - Macroscopic score				Extrusion		Deformation				
	**Tibia**		**Femur**		**Femoral**	**Tibial**	**Anterior area**	**Mid area**	**Posterior area**	**Length**	**Width**
**AnimalID**	*grader 1*	*grader 2*	*grader 1*	*grader 2*	*(mm)*	*(mm)*	*(% change with respect to the reference implant)*				
Implant 1	4	4	2	2	4.89	3.08	-0.2%	0.3%	-7.9%	1.46%	0.86%
Implant 2	3	3	2	2	2.99	2.99	-1.8%	-0.2%	-12.1%	2.52%	0.75%
Implant 3	4	3	2	2	3.62	4.80	-5.4%	-5.8%	-13.6%	2.49%	-0.19%
Implant 4	2	2	2	2	4.25	5.06	-0.9%	1.6%	-14.8%	2.13%	1.74%
Implant 5	3	2	2	2	2.16	2.93	-0.5%	0.2%	-11.5%	3.02%	1.27%
Implant 6	3	2	4	4	2.73	3.36	0.2%	0.8%	-12.3%	3.74%	0.99%
Implant 7	3	3	2	2	3.66	7.02	-4.3%	-4.9%	-14.1%	2.62%	-0.17%
Implant-control 1	2	2	2	2							
Implant-control 2	3	3	2	2							
Implant-control 3	1	1	2	2							
Implant-control 4	2	2	2	2							
Implant-control 5	3	2	2	2							
Implant-control 6	1	1	2	2							
Implant-control 7	1	2	3	3							
Sham 1	2	1	1	1	0.75	1.27					
Sham 2	1	1	1	1	1.36	0.78					
Sham 3	1	1	2	2	0.53	1.07					
Sham 4	2	2	2	2	1.32	0.69					
Sham 5	1	1	2	2	0.75	1.60					
Sham 6	2	2	2	2	0.88	1.36					
Sham-control 1	1	1	2	2							
Sham-control 2	2	2	2	2							
Sham-control 3	2	2	2	2							
Sham-control 4	3	2	2	2							
Sham-control 5	2	2	2	2							
Sham-control 6	2	2	2	2							

	Synovium - Histology score		Cartilage - Modified Mankin score					
			Tibia-inner		Tibia-outer		Femur	
**AnimalID**	*grader 1*	*grader 2*	*grader 1*	*grader 2*	*grader 1*	*grader 2*	*grader 1*	*grader 2*
Implant 1	2	5	21	23	21	8	16	19
Implant 2	3	7	22	18	11	11	10	10
Implant 3	6	8			25	24	13	10
Implant 4	4	2	21	19	15	17	10	6
Implant 5	3	6	17	19	12	13	20	20
Implant 6	7	6	9	7	12	9	12	9
Implant 7	6	12	18	18			15	13
Implant-control 1	1	6	15	17	9	9	8	2
Implant-control 2	5	6	20	19	12	11	9	8
Implant-control 3	2	1	20	17	9	6	6	4
Implant-control 4	0	2	19	19	5	5	6	6
Implant-control 5	3	7	15	14	7	7	9	2
Implant-control 6	6	3	12	10	5	6	5	3
Implant-control 7	4	3	15	19	8	5	7	3
Sham 1	5	6			4	5	5	2
Sham 2	5	8	18	30	5	4	9	9
Sham 3	4	2	19	20	9	5	5	1
Sham 4	4	2	19	18	7	7	6	8
Sham 5	3	6	19	18	6	5	4	2
Sham 6	3	2	21	19	9	4	7	1
Sham-control 1	5	6	20	20	5	4	11	3
Sham-control 2	3	4	16	15	8	6	4	1
Sham-control 3	2	3	20	19	7	5	5	2
Sham-control 4	2	3	17	19	7	6	7	5
Sham-control 5	3	8	17	18	8	5	6	4
Sham-control 6	3	2					7	0

	Cartilage - Affected Area score					
	**Tibia-inner**		**Tibia-outer**		**Femur**	
**AnimalID**	*grader 1*	*grader 2*	*grader 1*	*grader 2*	*grader 1*	*grader 2*
Implant 1	5	5	3	4	4	5
Implant 2	5	5	2	1	3	5
Implant 3	0	0	5	5	4	4
Implant 4	5	5	3	4	2	0
Implant 5	4	5	3	3	4	4
Implant 6	1	0	2	2	3	4
Implant 7	5	5	0	0	3	5
Implant-control 1	5	5	2	2	1	0
Implant-control 2	5	5	1	0	1	0
Implant-control 3	5	5	0	0	1	0
Implant-control 4	5	5	0	0	5	5
Implant-control 5	4	5	1	0	0	0
Implant-control 6	3	4	1	0	0	3
Implant-control 7	5	4	0	0	0	0
Sham 1	0	0	0	0	0	0
Sham 2	5	5	1	0	5	5
Sham 3	5	5	0	0	0	0
Sham 4	5	5	0	0	1	2
Sham 5	5	5	2	0	0	0
Sham 6	5	5	1	0	1	0
Sham-control 1	5	5	1	0	3	0
Sham-control 2	4	5	0	0	0	0
Sham-control 3	5	5	1	0	1	0
Sham-control 4	5	5	0	0	1	0
Sham-control 5	5	5	0	0	1	0
Sham-control 6	0	0	0	0	0	0
